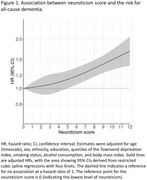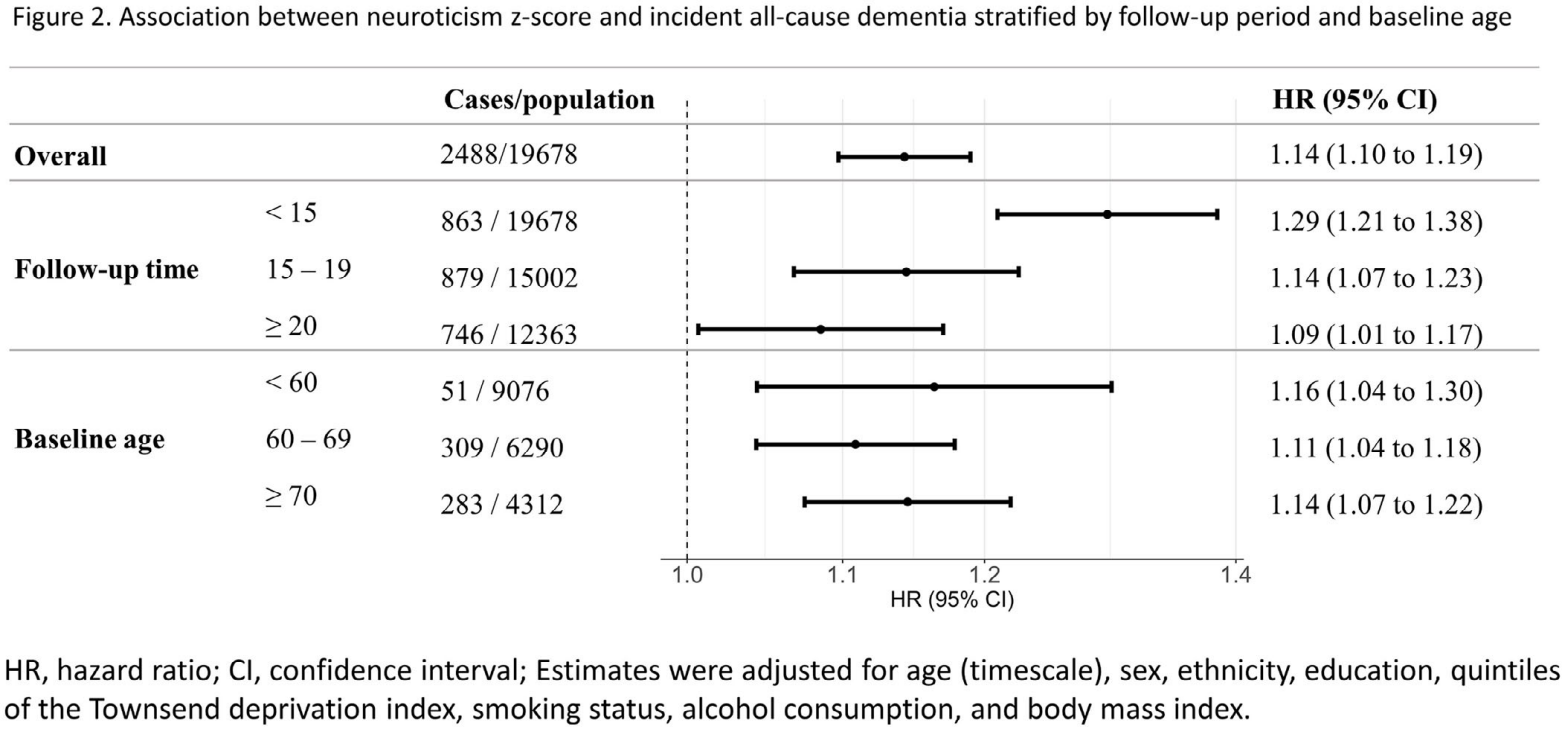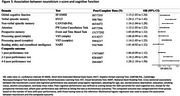# Association of neuroticism with incident dementia and cognitive function: 26 year follow‐up of EPIC‐Norfolk study

**DOI:** 10.1002/alz70860_104678

**Published:** 2025-12-23

**Authors:** Yaqing Gao, Robert Luben, Shabina Hayat, Najaf Amin, Cornelia M Van Duijn, David J Hunter, Thomas J Littlejohns

**Affiliations:** ^1^ University of Oxford, Oxford, Oxfordshire, United Kingdom; ^2^ University College London, London, England, United Kingdom

## Abstract

**Background:**

Several studies have reported an association between neuroticism and dementia. However, most measured neuroticism only in late life and had short follow‐up periods, raising concerns about reverse causality, as personality changes may occur in preclinical dementia. We aimed to address these limitations by examining this association in a large cohort with a wide baseline age range (40–80 years) and over 20 years of follow‐up, allowing for analysis of mid‐ and late‐life associations.

**Method:**

The EPIC‐Norfolk Study is a prospective cohort that recruited 30,445 men and women aged 39‐79 between 1993‐1997. 20,921 participants attended follow‐up between 1996‐2000, during which they completed a self‐reported neuroticism scale. Participants were followed through electronic linkage to hospital inpatient records, death registries, and mental health care data up to 2022 to ascertain all‐cause dementia. Cognitive tests were conducted between 2006 and 2011 in 8,585 participants. The association between neuroticism and dementia incidence was assessed using Cox proportional hazards models, while its association with cognitive performance was assessed using logistic regression. Both models were adjusted for age, sex, socioeconomic deprivation, education, smoking status, alcohol consumption, and body mass index.

**Result:**

Of 19,678 dementia‐free participants (mean [SD] age, 60.8 [9.3] years; 10,973 female [55.8%]), 2,488 developed dementia over a median follow‐up of 22.7 years. Higher neuroticism scores showed a dose–response relationship with increased dementia risk, with a 1 standard deviation increase in neuroticism associated with a 14% higher risk (adjusted hazard ratio 1.14 [95% confidence interval, 1.10‐1.19]). This association weakened over the follow‐up period but remained significant, even when restricted to participants followed for ≥20 years (1.09 [1.01‐1.17]). Consistent associations were observed across mid and late life: age <60 years at baseline (1.16 [1.04‐1.30]), 60‐70 years (1.11 [1.04‐1.18]), and ≥70 years (1.14 [1.07‐1.22]). Neuroticism was associated with deficits across multiple cognitive domains, with the strongest association observed for verbal memory. Higher neuroticism scores were associated with poor performance in a greater number of domains.

**Conclusion:**

High neuroticism levels in both mid‐ and late‐life were associated with long‐term dementia risk, and were associated with poor cognitive function in individuals without dementia.